# Coronavirus disease 2019 presenting as psychosis: a case report

**DOI:** 10.1186/s13256-022-03349-z

**Published:** 2022-04-22

**Authors:** Nyanyuie Kodjo Lovi, Selase Kofi Kekrebesi, Mary Osei, Eugene Yeboah

**Affiliations:** 1 Department of Internal Medicine, Accra Medical Centre, Accra, Ghana; 2 Accra Medical Centre, Accra, Ghana

**Keywords:** Case report, COVID-19, Psychosis

## Abstract

**Background:**

The coronavirus disease 2019 syndrome typically consists of respiratory symptoms and other general nonspecific symptoms. Psychotic manifestations of coronavirus disease 2019 attributable to severe acute respiratory syndrome coronavirus 2 infection are seldom reported. We report a case of coronavirus disease 2019 in a young West African male who had no known risk factors of psychiatric illness or past history of psychiatric disease presenting with acute psychosis.

**Case presentation:**

Our patient, who was a young West African male, presented without the typical respiratory symptoms of coronavirus disease 2019 and also without a background history of psychiatric illness or any other significant stressors in his past or present social history. He had acute onset of psychotic symptoms consisting of visual and auditory hallucinations, delusions of persecution, and lack of insight. He was admitted and managed with antipsychotic medication and mood stabilizer. His laboratory workup was normal except for positive coronavirus disease 2019 polymerase chain reaction and his liver enzymes, which showed elevated gamma glutamyl transferase, a finding consistent with coronavirus disease 2019. His head computed tomography scan was also normal. The patient made a gradual recovery from his psychotic symptoms, with gain of insight 7 weeks after onset of symptoms, at which time his coronavirus disease 2019 test came back negative along with other laboratory parameters. He returned to work 12 weeks after his presentation and has been performing well.

**Conclusion:**

Psychosis can be a primary presenting symptom in patients with coronavirus disease 2019, including those without respiratory symptoms.

## Background

The novel coronavirus disease 2019 (COVID-19) caused by severe acute respiratory syndrome coronavirus 2 (SARS-CoV2) originated in December 2019 in Wuhan, China [1]. It has since spread rapidly worldwide, causing morbidity and mortality in its wake. In March 2020, the World Health Organization (WHO) declared the COVID-19 outbreak to be a pandemic [2].

It is widely known that patients infected with the novel coronavirus SARS-CoV2 present respiratory tract symptoms such as anosmia, rhinorrhea, sore throat, cough, fever, and difficulty in breathing [3]. Other symptoms not related to the respiratory system include diarrhea, myalgia, and headaches [4]. Although there has been extensive literature on other organ systems affected by COVID-19, cases of psychiatric illness have not received due attention [5].

A few cases of exacerbation or precipitation of psychotic episodes among patients with preexisting psychiatric conditions or who have risk factors for psychiatric illness have been reported [6].

We report a case of a patient with no known psychiatric conditions who presented to the consulting room with acute onset of hallucinations and delusions and tested positive for COVID-19. Apart from these psychiatric symptoms, the patient did not present with the usual well-known symptoms and signs of COVID-19 such as fever, cough, dyspnea, and hypoxemia. We believe our report will add up to the existing knowledge on the clinical manifestations of COVID 19 and raise awareness among physicians for prompt recognition and management of the disease.

## Case presentation

Our patient, who was a 35-year-old West African male born to Ghanaian parents, barged into the outpatient consulting room in the company of two family members while saying in a loud voice “I am a man of God, I am rich, and a strong man, and all those with ill intention towards me can do nothing to me.” He kept pacing back and forth with much energy in the consulting room. A week prior to this presentation, his family reported that the patient had episodes of talkativeness, incoherent speech, visual hallucinations, auditory hallucinations, and insomnia for several days.

This was the first episode of such bizarre behavior in his life as reported by his family. He had no history of head injury, substance abuse, childhood abuse, or learning disabilities. The patient had no known chronic medical condition, neither was he on any long-term medications. He was not vaccinated against COVID-19 since at the time of presentation vaccination was yet to be rolled out widely in Ghana.

There was no family history of psychiatric illness on either the paternal or maternal side of his family. Both of his parents were alive and well with no known chronic medical illness. Likewise, his siblings were all alive and did not have any known chronic medical illness.

The patient grew up in a gold mining community, where there was often illegal mining with mercury, and spent most of his life in the community. He left the community for about 5 years to pursue tertiary education but returned to the community to take up a permanent working position as a mechanical engineer. He had not reported any unusual work-related stress previously. Apart from his work, he had other businesses that took him to surrounding communities. At the time of his illness, there were reports of community spread of COVID-19 in those communities. However, the patient did not express any anxiety regarding the COVID-19 pandemic. He was also mostly adherent to the COVID-19 risk reducing guidelines which involved the  use of face masks, regular hand washing, and physical distancing. He was a religious person, married, with no report of a stressful marital life. He had never engaged in small-scale or illegal mining previously. The source of water for the community he grew up in was via pipe supplied by the national water company.

### Examination

The patient looked generally well and was afebrile. There were no stigmata of chronic illness. He was afebrile, anicteric, acyanosed, mildly dehydrated, and did not have any lymphadenopathy. His general physical examination was normal.

He had normal respiratory rate, adequate symmetrical chest expansion, normal tactile fremitus, normal percussion notes, and vesicular breath sounds. His blood pressure was within the normal range at 135/80 mmHg; pulse was 88 bpm at presentation. His heart sounds were normal with no added sounds. There was no evidence of peripheral vascular disease. The abdomen and musculoskeletal examinations were also normal.

Neurologically he was oriented in time, place, and person, Kernig’s sign was negative, and both pupils were equal and reactive to direct and consensual light reflex.

He was inappropriately dressed and looking disheveled. His speech was clear, with normal accent, rate, tone, and no stuttering. His speech was of increased volume, but there was no latency. He was restless and looked agitated but could establish normal eye contact. There were no mannerisms, stereotypes, or posturing. His thought was circumstantial without any suicidal ideation. He was grandiose, believing he was a prophet of God. He had delusions of persecution as he believed there were people with evil intention all around who desired to harm him. He had both auditory and visual hallucinations as he reported that he had heard gunshots and could see a mob coming after him. During the examination, he broke down into tears suddenly, without a tangible explanation. His short- and long-term memories were unimpaired, and he did not have insight into his condition.

### Investigation

The investigations requested were a complete blood count, malaria profile, renal function test, liver function test, glycated hemoglobin, hepatitis B surface antigen, hepatitis C antibodies, COVID-19 PCR, and head CT scan.

COVID-19 screen was added because his presentation was unusual with no risk factors for psychotic behavior. Additionally, he was not immunized against COVID-19 and was probably exposed to the infection because he often traveled through communities with recorded outbreaks of COVID-19 before his presentation.

Mercury levels were requested on account of possible exposure as a resident in a mining community in which the practice of using mercury for mining was rampant. However, this laboratory analysis could not be done because this was a specialized investigations and could not be run due to limitations in laboratory resources at the given time period.

### Laboratory results

**Fig. 1:**
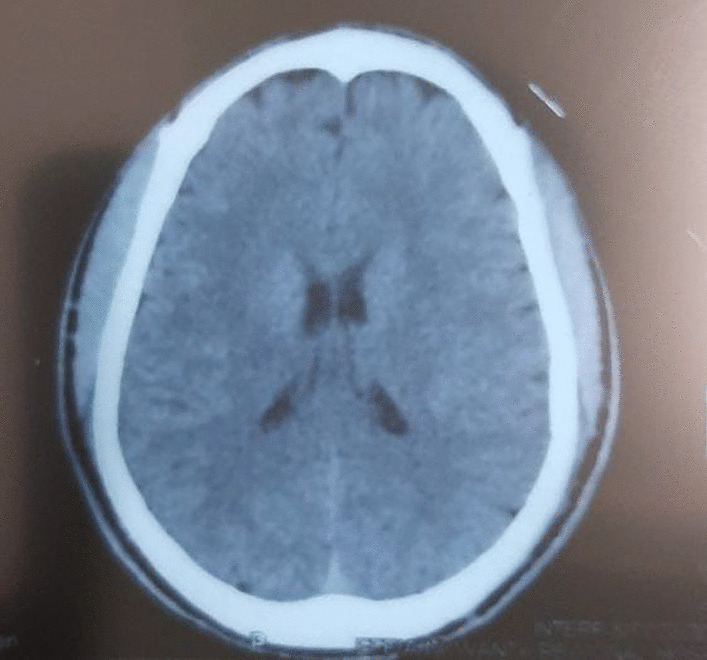
Head Computed Tomography scan of patient

Of his initial labs requested, urinalysis showed proteinuria+ and glycosuria 4+. A repeat of his urinalysis 3 days later was normal. Reasons for the glucosuria was attributed to the acute stress the patient was experiencing as well as the use of chlorpromazine to sedate the patient (Tables 1, 2, 3, 4, and 5; Fig. 1).

**Table 1:** Full blood count results of the patient

RBC	4.34/pl L	4.5–6.5
Hb (Hemoglobin)	13.3 g/dL	13.0–18.0
Hematocrit	0.41 L/L	0.40–0.54
MCV	95 fL	76–99
MCH	30.6 pg	26–33
MCHC	32.3 g/dL	30–37
RDW	11.7%	11.0–16.0
Platelets	186 × 10^9^/L	150–450
WBC only	6.6 × 10^9^/L	4.0–12.0
Neutrophils	62.0%	
Lymphocytes	27.5%	
Monocytes	8.9%	
Eosinophils	1.3%	
Basophils	0.3%	

*RBC* red blood cell count,* MCV* mean copuscular volume,* MCH* mean copuscular haemoglobin,* MCHC* mean copuscular haemoglobin concentration,* WBC* white blood cell

**Table 2:** Malaria and Typhoid test results of the patient

Malaria smear (thin, thick)	Both negative
Malaria antigen	Negative
Typhoid antibody	Negative for IGM and IGG

*IGM* immunoglobulin type M,* IGG* immunoglobulin type G

**Table 3:** Liver function test results of the patient

S-Bilirubin (total)	26 umol/L	3.42–20.5
S-Bilirubin conjugated	5 umol/L	< 5
S-Alkaline phosphatase	49 IU/L	40–1
S-G-Glutamyl transferase	81 IU/L	< 55
S-Alt	42 IU/L	0–41
S-Ast	29 IU/L	0–40
S-Albumin	45 g/L	39.7–49.5
S-Total protein	77 g/L	64–83

*S* serum

**Table 4:** Renal function test results  and HBA1C of the patient

Renal function test		
	Result	
S-Sodium	136 mmol/L	136–145
S-Potassium	3.9 mmol/L	3.5–5.1
S-Chloride	101 mmol/L	98–107
Total CO_2_ (bicarbonate)	Serum 26 mmol/L	22–29
S-Urea	3.2 mmol/L	2.1–7.1
S-Creatinine	81 umol/L	62–106
eGFR-(CKD-EPI)	> 89 mL/min	
HBA1C	4.5%	4.8–5.9

*eGFR* estimated glomerular filtration rate,* CKD-EPI* chronic kidney disease epidemiology collaboration,* HBA1c* glycated haemoglobin 1c,* S* serum

**Table 5:** Urinalysis of the patient

Blood/hemoglobin	Not detected
Glucose	+++
Ketones	Not detected
Leukocyte esterase	Not detected
Nitrite	Negative
Protein	+
Specific gravity	1.015
Urobilinogen	Not detected
Bacteria	Not observed
Casts	Not observed
Crystals	Not observed
Erythrocytes	Not observed
Squamous epithelium	Not observed
Yeast, hyphae	Not observed

His serum GGT was raised to almost twice the upper limit of normal, and his total bilirubin was marginally elevated. However, other liver enzymes and albumin were normal.

COVID-19 antigen test done using a lateral flow test kit was positive and followed up with RT-PCR using a nasopharyngeal swab sample, which was also positive for COVID-19. As per the hospital’s COVID 19 testing protocol at the time, patients with suspected COVID-19 infection were initially screened with a rapid test and those found positive would have a confirmatory PCR done. Additional markers of inflammation such as LDH, d-dimers, and ferritin, used in part to classify the severity of the infection and also for prognostic purposes [7], were not requested following the positive test result of our patient because of cost to the patient. We felt that, although these tests were useful for determining the severity of COVID-19, we could also judge the severity of the patient’s illness from the clinical presentation, physical examination, and also from the basic laboratory parameters [8].

The head CT scan was reported as normal by the radiologist.

## Differential diagnosis

In view of the normal physical examination, baseline investigations, as well as absence of any pointers to an organic cause of the patient’s presentation in his history, the following differential diagnoses were entertained:

1. Brief psychotic disorder
2. Bipolar I disorder
3. Mercury toxicity
4. COVID-19-induced psychosis

Brief psychotic disorder was considered as the first diagnosis at the initial visit because the patient had new-onset psychotic symptoms and was within the 1-month period designation for the diagnosis.

Bipolar I disorder was considered as an alternate diagnosis because the patient had symptoms that were compatible at presentation. Clearly, the patient was euphoric, hyperactive, and talkative, in line with this differential.

COVID-19 infection was considered a possible cause of the patient’s symptoms because his test was positive at the time and there was emerging evidence of a possible association between COVID-19 and psychosis [6].

The possibility of heavy metal poisoning was entertained because the patient had lived within a community with illegal mining for a significant period of time. However, this was not a likely diagnosis since the patient did not have somatic symptoms indicative of the diagnosis. Furthermore, this differential was considered to be ruled out following the recovery of the patient without treatment specific to mercury poisoning. The head CT scan did not suggest any space-occupying lesions, ischemic changes, or depositions.

## Treatment

The patient was admitted and was initially given an intramuscular injection of chlorpromazine. He was later evaluated by a psychiatrist and started on oral olanzapine 10 mg daily and oral carbamazipine 500 mg daily. His care was then continued on an outpatient basis because the patient was more cooperative with his treatment in the care of his immediate family. He was followed up with phone calls and encouraged to be compliant with medications. Additional support of his care was provided by a general practitioner on an outpatient basis. Educational support was also given to the family regarding his care.

## Outcome and follow-up

**Fig. 2:**
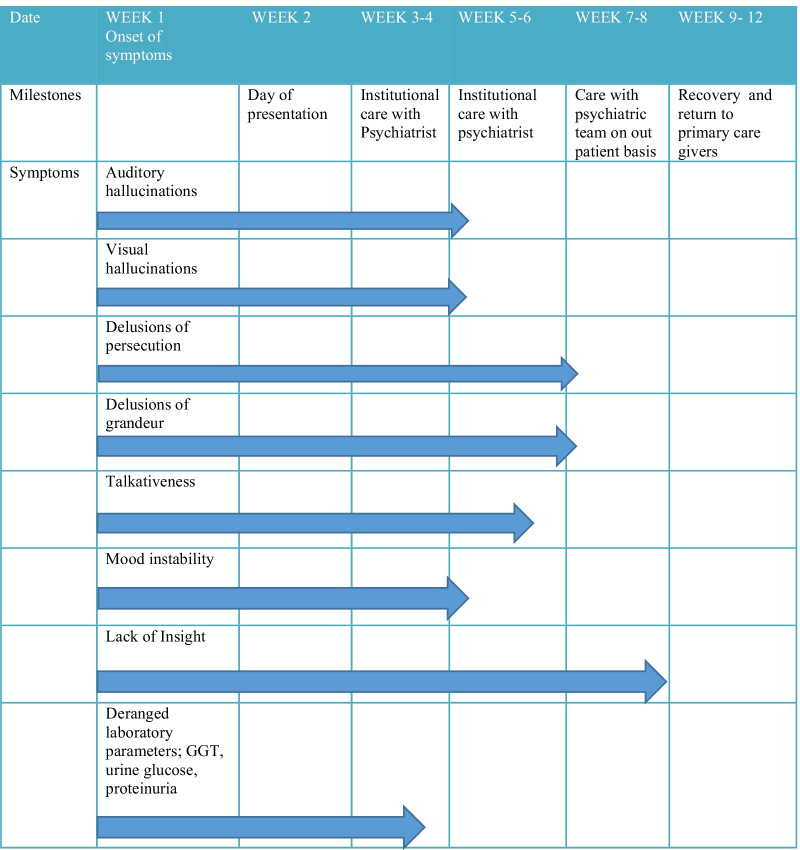
Chart showing timelines from onset of symptoms to resolution of symptoms

The patient had a steady recovery after the initiation of medications. The resolution of his symptoms was preceded by the resolution of his laboratory parameters about 4 weeks after the onset of his symptoms. His symptoms resolved over a 7-week period starting with the hallucinations and finally a gain of insight into his condition 7 weeks after his initial presentation (Fig. 2). He clearly recollected all the events that took place during his presentation and management. After a final evaluation by the medical team, and later an independent assessment by the psychiatrist, the patient was found to have recovered fully, at which time his COVID-19 PCR was negative and he was fit to return to work. His diagnosis was modified to COVID-19-associated acute and transient psychotic disorder after his illness persisted beyond the 1-month period defined for brief psychotic disorder. He was recalled to work 12 weeks after the illness onset and has been performing satisfactorily at his post.

## Discussion

COVID-19 infection, although primarily a respiratory tract infection, has been shown to present with other systemic manifestations [9]. Although the coronavirus specifically infects the respiratory system, it also has contributed to psychological illnesses such as depression, anxiety, and sleep disorders [10, 11]. Psychiatric symptoms associated with COVID-19 have also been reported, mostly among patients with preexisting psychiatric illness presenting with a relapse of their condition owing to the COVID-19 infection [6]. However, a handful of reports of new-onset psychiatric manifestations in association with severe COVID-19 have been made [6]. In this case report, the patient did not have any previous records of psychiatric illness and was found to have asymptomatic COVID-19 infection at the time of presentation.

The initial diagnosis considered for the patient was brief pyschotic disorder. However, after the first month post onset of the patient’s symptoms had elapsed, brief psychotic disorder was no longer considered as the primary diagnosis as according to the ICD-10 definition of brief psychotic disorder, the symptoms should have resolved within a month of onset. This then made acute and transient psychotic disorder the next probable diagnosis [12]. The patient’s symptoms fit the diagnosis as his symptoms resolved within 3 months of onset. What was new about this patient was the associated COVID-19 infection.

Previous case reports of COVID-19 in association with new-onset psychiatric manifestations have postulated that COVID-19 could be associated with psychiatric illness by unmasking patients having an undiagnosed underlying history of psychiatric disease or that the COVID-19 infection could be a direct etiological factor for new-onset psychiatric symptoms [6]. These case reports frequently involved patients who were often above the age of 60 years, had other comorbidities such as hypertension and diabetes, and often had moderate to severe COVID-19 respiratory symptoms [6].

Our patient at the time of diagnosis of COVID-19 was young, did not have any known medical comorbidities, and did not have any respiratory symptoms or other systemic symptoms. Additionally, parameters such as full blood count, white cell count, platelet count, and albumin were all well within the normal ranges. However, GGT and total bilirubin were elevated, suggesting cholestasis, a feature in frequent association with systemic inflammation [13]. The albumin-to-GGT ratio was 0.5, supporting an ongoing inflammatory response [14]. Elevated liver enzymes are a common finding in COVID-19 and reflect both the local and systemic inflammatory response to the viral infection [15].

Although traditional markers of inflammation were not requested, we deemed the deranged parameters as surrogate markers for inflammation, which we followed up with. The timing of their resolution, about 4 weeks post onset of symptoms, also coincided with the onset of resolution of the patient’s psychiatric symptoms. The relationship between the timing of the resolution of the surrogate markers of systemic inflammatory and the psychotic symptoms was similar to that of other case reports with a similar patient profile to ours [6, 16].

It is our hypothesis that the patient might have experienced a transient systemic inflammation from the COVID-19 infection with subsequent psychotic manifestations and might have presented to us at a time when his laboratory parameters had almost completely normalized, keeping in mind that the symptoms he presented with had been ongoing for a at least a week before the index hospital visit.

A second possibility was that the patient may have had undiagnosed underlying psychiatric disease or risk factors and subsequently presented with psychosis due to the stress and transient inflammation associated with COVID-19 infection.

With the head CT scan of our patient being normal, a cerebral ischemic event was less likely to be the explanation for the patient’s manifestation. We do however concede that a normal head CT scan did not rule out any other brain lesions that could be identified using better imaging modalities such as MRI [17]. The exclusion of brain lesions, particularly ischemic events, was important in our patient because evidence has been shown that the SARS-CoV2 virus may induce hypercoagulability and thrombosis in various organs, including the lungs, liver, kidneys, heart, muscles, gastrointestinal system, and brain [18–20].

Currently, no established clinical guidelines exist on the management of patients with neuropsychiatric manifestation of SARS-CoV2 infection. Although clinical trials for treatment have been ongoing, the focus has been on the more dramatic and life-threatening manifestations such as acute respiratory distress. We managed our patient conservatively by providing only supportive care. We did not make use of any investigational treatments. The patient was tolerant to antiseizure medication used for the purpose of mood stabilization. The patient was less tolerant of the antipsychotic medication due to the severe drowsiness he experienced when he took it.

Our patient was considered to have made a full recovery enabling him to return to work 12 weeks after onset of his symptoms. This was consistent with a review of other case reports in which most of the patients reportedly made full functional recovery after 3 months following the initial symptoms [6].

## Conclusion

Psychosis can be a primary presenting symptom in patients with COVID-19. Such patients may not have respiratory symptoms at the time of presentation.

## Data Availability

Authors can confirm that all relevant data are included in the article.

## Abbreviations

COVID-19: Coronavirus disease 2019
SARS-CoV2: Severe Acute Respiratory Syndrome Coronavirus 2
PCR: Polymerase Chain Reaction
GGT: Gamma Glutamyl Transferase
CT: Computed Tomography
MRI: Magnetic Resonance Imaging
